# Exploring shared microRNA profiles in liquid-based cytology and plasma as biomarkers of high-grade intraepithelial lesions

**DOI:** 10.1038/s41598-025-30514-3

**Published:** 2025-12-02

**Authors:** Stéphanie Calfa, Ana Julia Aguiar de Freitas, Rhafaela Lima Causin, Welinton Hirai, Júlio César Possati-Resende, Ricardo dos Reis, Rui Manuel Reis, Márcia Maria Chiquitelli Marques

**Affiliations:** 1 Molecular Oncology Research Center, Teaching and Research Institute, Barretos Cancer Hospital, Barretos, SP 14784-400 Brazil; 2 Department of Prevention, Barretos Cancer Hospital, Barretos, Brazil; 3 Gynecologic Oncology Department, Barretos Cancer Hospital, São Paulo, Brazil; 4 Life and Health Sciences Research Institute (ICVS), School of Medicine, University of Minho, Braga, Portugal

**Keywords:** High-grade cervical intraepithelial lesion, microRNAs, Liquid biopsy, Biomarker, miR-339-3p, Biomarkers, Cancer, Computational biology and bioinformatics, Molecular biology, Oncology

## Abstract

High-grade cervical intraepithelial lesions (CIN 2/3) are critical targets for early detection in cervical cancer prevention. MicroRNAs are stable, tissue-specific molecules involved in cancer-related pathways and can be detected in minimally invasive samples, making them suitable for use in the context of liquid biopsies. This exploratory study aimed to identify differentially expressed microRNAs in paired liquid-based cytology (LBC) and plasma samples from women with CIN 2/3, and to evaluate miR-339-3p as a potential biomarker. Samples from 70 women (35 cases with histologically confirmed high-grade lesions and 35 age-matched controls with normal cytology and negative HPV tests) were analyzed using a commercial microRNA panel on the NanoString platform. We identified 57 dysregulated microRNAs in LBC samples and 33 in plasma, with four shared between both matrix types. Among these, miR-339-3p was the only one consistently upregulated and significantly associated with case status. In LBC samples, the area under the curve was 0.65; in plasma, 0.64. Pathway analysis suggested its involvement in apoptosis-related pathways, including PI3K-Akt and p53. Moreover, this study highlights the value of integrating microRNA profiling across local and systemic samples to advance biomarker discovery in cervical cancer.

**Supplementary Information:**

The online version contains supplementary material available at 10.1038/s41598-025-30514-3.

## Introduction

Cervical cancer represents a public health issue, standing as the fourth most common cancer among women worldwide^1^ and the third most prevalent cancer in Brazil^2^. Persistent infection with high-risk human papillomavirus (HPV) types is the primary cause of cervical carcinogenesis, causing the development of precursor lesions and invasive cancer^3^. High-grade cervical intraepithelial lesions (CIN 2 and CIN 3) are precursor lesions with a risk of progression to invasive cancer if left untreated^4^. Therefore, early detection and treatment of these lesions are essential to prevent cancer progression^5^.

Although Pap smears and HPV testing have improved the detection of cervical abnormalities, these methods still have limitations in terms of sensitivity, specificity, and inter-laboratory reproducibility^6,7^. Considering this, there is an increasing interest in developing novel biomarkers that could improve risk stratification, enhance diagnostic accuracy, and support better clinical decision-making^8^.

In recent years, liquid biopsy (LB) has been established as a minimally invasive technique capable of capturing the molecular characteristics of tumors through the analysis of circulating tumor-derived components in body fluids, such as blood, saliva, urine, and feces^9^. By detecting circulating tumor cells, cell-free DNA, cell-free RNA, and exosomes, LB offers new opportunities for early detection, prognostic, and therapeutic monitoring^10^. Specifically, plasma and liquid-based cytology (LBC) samples have become the usual matrices for identifying biomarkers in this context, particularly in cervical precursor lesions^11,12^.

Among the various molecular targets explored, microRNAs (miRNAs) are well-known for their important role in gene regulation and their stability in biological fluids^13^. These small non-coding RNAs influence essential biological pathways, as proliferation, differentiation, and apoptosis, which are deeply implicated in cancer development and progression^14^. Their stability across different sample types and disease-specific expression profiles makes them promising candidates for early diagnostic strategies^15^. Investigating miRNAs detectable in both LBC and plasma samples could provide essential insights into the mechanisms driving cervical lesion progression^11,14^. Moreover, identifying consistently present miRNAs across different matrices is particularly valuable for clinical applications, offering more robust and flexible diagnostic tools^13^. To our knowledge, no previous studies have directly compared miRNA expression between LBC and plasma samples.

Considering these points, after identifying the differentially expressed miRNAs shared between LBC and plasma samples, miR-339-3p was selected for further investigation due to its consistent upregulation and association with case status in both matrices. Previous studies suggest that miR-339-3p may act as a tumor suppressor, involved in pathways related to invasion and metastasis across several cancer types^16–18^. However, its specific role in cervical carcinogenesis, particularly in the progression of precursor lesions, remains poorly understood and requires further investigation.

In this study, we aimed to identify miRNAs differentially expressed in LBC and plasma samples from patients with CIN 2/3. Four candidates were identified among the miRNAs commonly found in both sample types (miR-339-3p, miR-520a-5p, miR-619-3p, and miR-330-3p). Of these, miR-339-3p was selected based on differential expression analyses for subsequent evaluation regarding its association with lesion progression and potential involvement in relevant biological pathways.

## Results

### Clinical-pathological and epidemiological characteristics of the study population

The main epidemiological and clinicopathological features of both case and control groups are summarized in Table 1. No statistically significant differences between groups for age (*p* = 0.61), confirming the expected similarity due to age matching, with a mean age of 36 years. No significant differences were observed for smoking status, alcohol consumption, menopausal status, or HPV vaccination. However, the groups differed significantly in the use of hormonal contraceptives (*p* = 0.015). In the cases, 77.2% had HSIL as the cytology result, 77.10% had a consolidated diagnosis of CIN 3, and 45.7% had infections with other high-risk HPV types.

**Table 1:** Descriptive and statistical analysis of epidemiological and clinicopathological characteristics of the study participants (case and control groups).

Characteristics	Cases (*n* = 35)	Controls (*n* = 35)	*p*-value
Age (years old)			
Mean (SD)	36.71 (8.89)	36.57 (8.97)	0.61^a^
Median (min-max)	36.00 (25–55)	35.00 (25–56)	
	n (%)	n (%)	
Smoking			
Yes	7 (20.00)	3 (8.60)	0.4^b^
No	26 (74.30)	31 (88.60)	
Former smoker	2 (5.70)	1 (2.90)	
Alcohol consumption			
Yes	25 (71.40)	24 (68.60)	0.053^b^
No	4 (11.40)	10 (28.60)	
Former consumption	6 (17.10)	1 (2.90)	
Menopausal status			
Pre-menopause	30 (85.70)	30 (85.70)	> 0.9^b^
Post-menopause	5 (14.30)	5 (14.30)	
Use of hormonal contraceptives			
Never	1 (2.90)	8 (22.90)	**0.015** ^b*^
Currently	17 (48.60)	9 (25.70)	
Former	17 (48.60)	18 (51.40)	
HPV vaccine			
Yes	3 (8.60)	6 (17.10)	0.5^b^
No	32 (91.40)	29 (82.90)	
Cytology			
ASC-US	4 (11.40)	−	**< 0.001** ^b*^
ASC-H	2 (5.70)		
LSIL	–		
HSIL	27 (77.20)		
Negative	2 (5.70)	35 (100)	
Consolidated diagnosis			
CIN 2	4 (11.40)	−	**< 0.001** ^b*^
CIN 2/3	4 (11.40)		
CIN 3	27 (77.10)		
HPV status			
HPV 16	9 (25.70)	–	**< 0.001** ^b*^
HPV 18	2 (5.70)		
Other hr-HPV types	16 (45.70)		
HPV 16 + other high-risk types	5 (14.30)		
HPV 18 + other high-risk types	−		
Negative	3 (8.60)	35 (100)	

SD, standard deviation; Min, minimum; Max, maximum; ASC-US, atypical squamous cells of undetermined significance; ASC-H, atypical squamous cells—cannot exclude high-grade intraepithelial lesions; HSIL, high-grade squamous intraepithelial lesions; LSIL, low-grade squamous intraepithelial lesions; Consolidated diagnosis: the highest grade result (biopsy or anatomopathological), CIN, cervical intraepithelial lesion; hr-HPV, high-risk human papillomavirus.

^a^Mann–Whitney test.

^b^Chi-square test, *p < 0.05.

Significant values are in [bold].

### miRNA differential expression analysis

Considering a significance threshold of *p* < 0.05, in the LBC samples, a total of 57 differentially expressed miRNAs were identified (42 were upregulated and 15 were downregulated), while in the plasma samples, 33 miRNAs were found to be differentially expressed (10 upregulated and 23 downregulated). A complete list of these miRNAs is provided in the supplementary table (Tables S1 and S2).

**Fig. 1:**
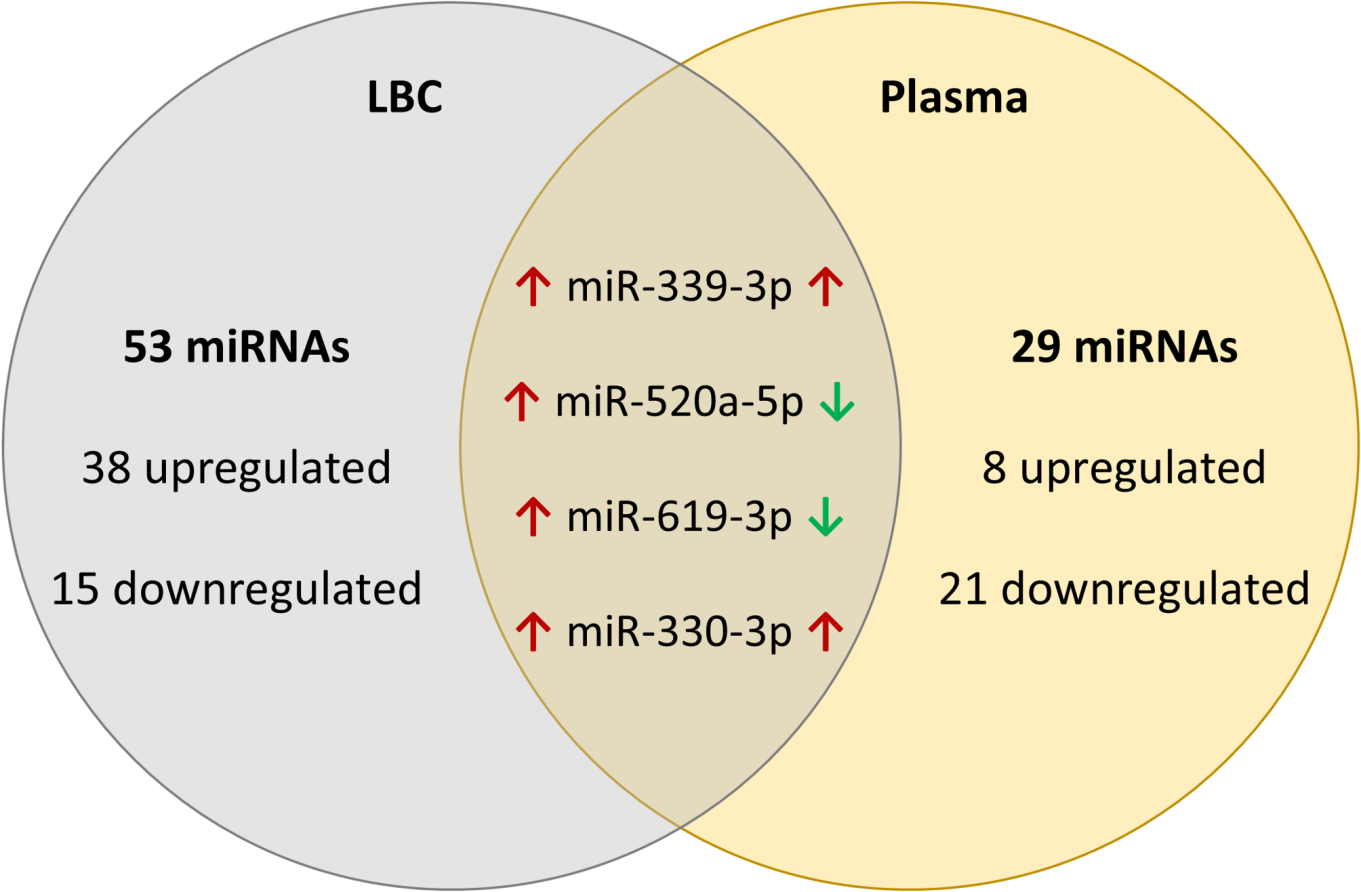
Venn diagram illustrating the overlap of differentially expressed miRNAs between case and control groups in LBC and plasma samples. Red upward arrows (↑) indicate overexpressed miRNAs, while green downward arrows (↓) represent underexpressed miRNAs.

We compared the 33 miRNAs identified in plasma with the 57 miRNAs detected in LBC to investigate whether any differentially expressed miRNAs were shared between the two matrices (Fig. 1). We observed four miRNAs commonly differentially expressed in both matrices: miR-339-3p, miR-520a-5p, miR-619-3p, and miR-330-3p.

**Fig. 2:**
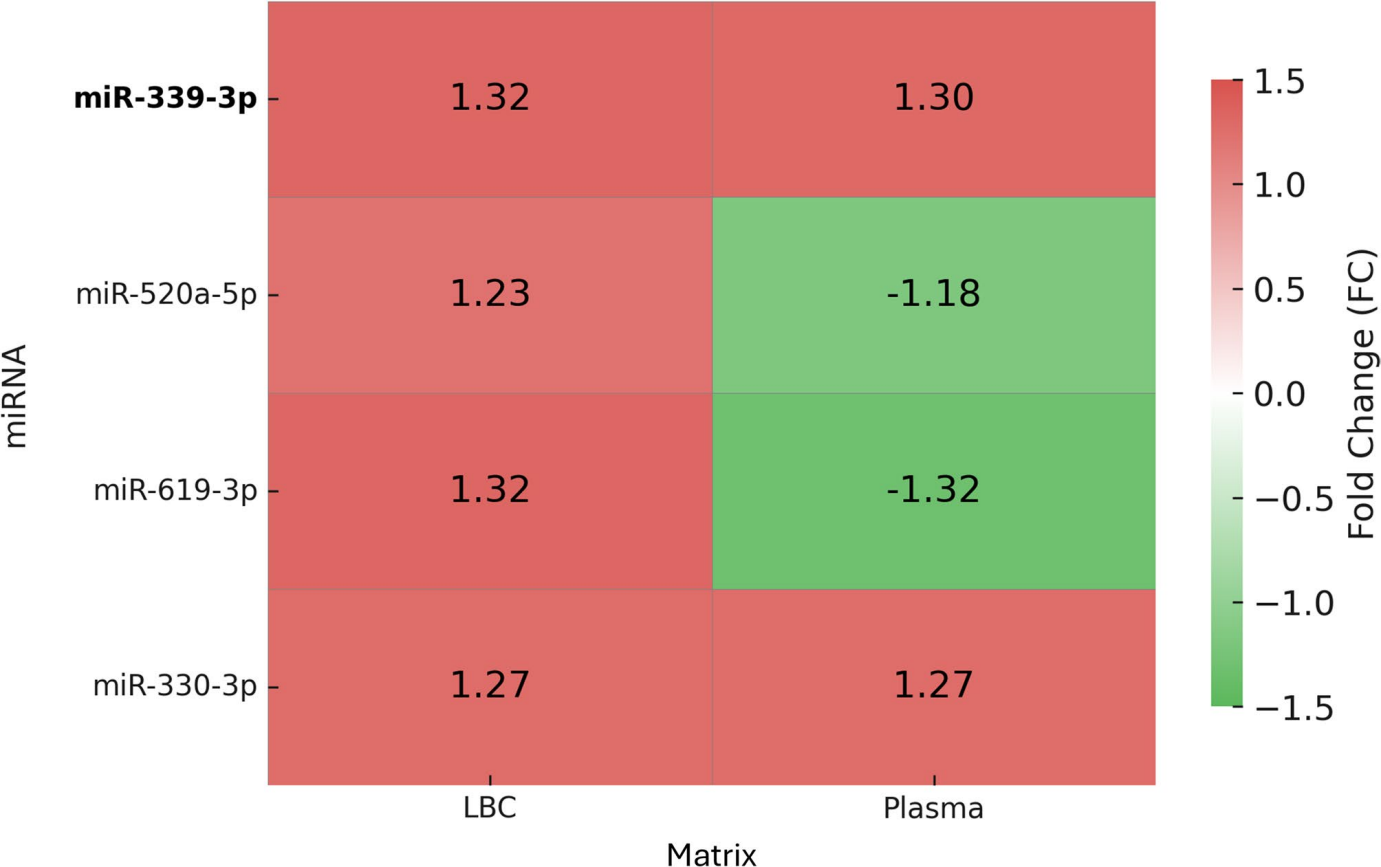
Expression profile of shared differentially expressed miRNAs in LBC and plasma samples.

To explore the expression patterns of these shared miRNAs across both matrices, a heatmap was generated using the fold change values observed in LBC and plasma (Fig. 2). While all four miRNAs were upregulated in LBC, their expression profiles diverged in plasma: miR-520a-5p and miR-619-3p were downregulated, whereas miR-339-3p and miR-330-3p remained upregulated.

### Multivariate regression-based miRNA selection

Multivariate logistic regression analyses were performed separately for LBC and plasma samples to assess the association between miRNA expression levels and use of hormonal contraceptives. A backward selection method was applied to identify the most relevant predictors (Table 2).

**Table 2:** Multivariate logistic regression analysis of shared MiRNAs in LBC and plasma, with epidemiological variables significantly different between case and control groups, using backward selection.

Characteristic	Variables	Multivariate logistic (LBC)			Multivariate logistic (plasma)		
		OR	95%CI	p-value	OR	95%CI	p-value
miR-339-3p	Underexpression	−	−	−	−	−	−
	Overexpression	4.78	1.23–22.2	0.031*	6.36	1.54-33.0	0.016*
miR-520a-5p	Underexpression	−	−	−	−	−	−
	Overexpression	0.70	0.19–2.57	0.6	3.18	0.79-15.0	0.12
miR.619.3p	Underexpression	−	−	−	−	−	−
	Overexpression	1.30	0.35–5.27	0.7	10.8	2.88–51.7	0.001**
miR-330-3p	Underexpression	−	−	−	−	−	−
	Overexpression	1.07	0.25–4.77	**>** 0.9	0.20	0.04–0.79	0.031*
Hormonal contraceptive	Never	−	−	−	−	−	−
	Currently	17.9	2.18–428	0.022*	13.4	1.46–332	0.044*
	Former	7.65	1.00-172	0.094	11.4	1.31–278	0.057

OR, odds ratio; CI, confidence interval.

*p < 0.05; **p < 0.01; ***p < 0.001.

Significant values are in [bold].

For the LBC samples (Table 2), miR-339-3p overexpression remained significantly associated with the case group (OR 4.78; 95% CI 1.23–22.2; *p* = 0.031). The current use of hormonal contraceptives (OR 17.9; 95% CI 2.18–428; *p* = 0.022) was also significantly associated with higher odds of being in the case group.

In plasma samples (Table 2), three miRNAs remained in the final model. miR-339-3p (OR 6.36; 95% CI 1.54–33.0 *p* = 0.016) and miR-619-3p (OR 10.8; 95% CI 2.88–51.7; *p* = 0.001) were significantly overexpressed in the case group, while miR-330-3p was underexpressed (OR 0.20; 95% CI 0.04–0.79; *p* = 0.031). The current use of hormonal contraceptives was again associated with increased odds of being in the case group (OR 3.4; 95% CI 1.46–332; *p* = 0.044). To verify the stability of the model, a 5-fold cross-validation was conducted, as shown in Supplementary Tables S4 and S5. The overall agreement (accuracy) between predicted and actual classes was 61.43% for both LBC and plasma.

Among the four miRNAs commonly differentially expressed in both LBC and plasma samples, miR-339-3p was the only one that remained significant in the final multivariate logistic regression models for both matrices. In plasma, miR-619-3p and miR-330-3p also showed significant associations with case status. Given its consistent differential expression and presence in both regression models, miR-339-3p was selected for further analysis.

### Exploratory analysis of miR-339-3p

**Fig. 3:**
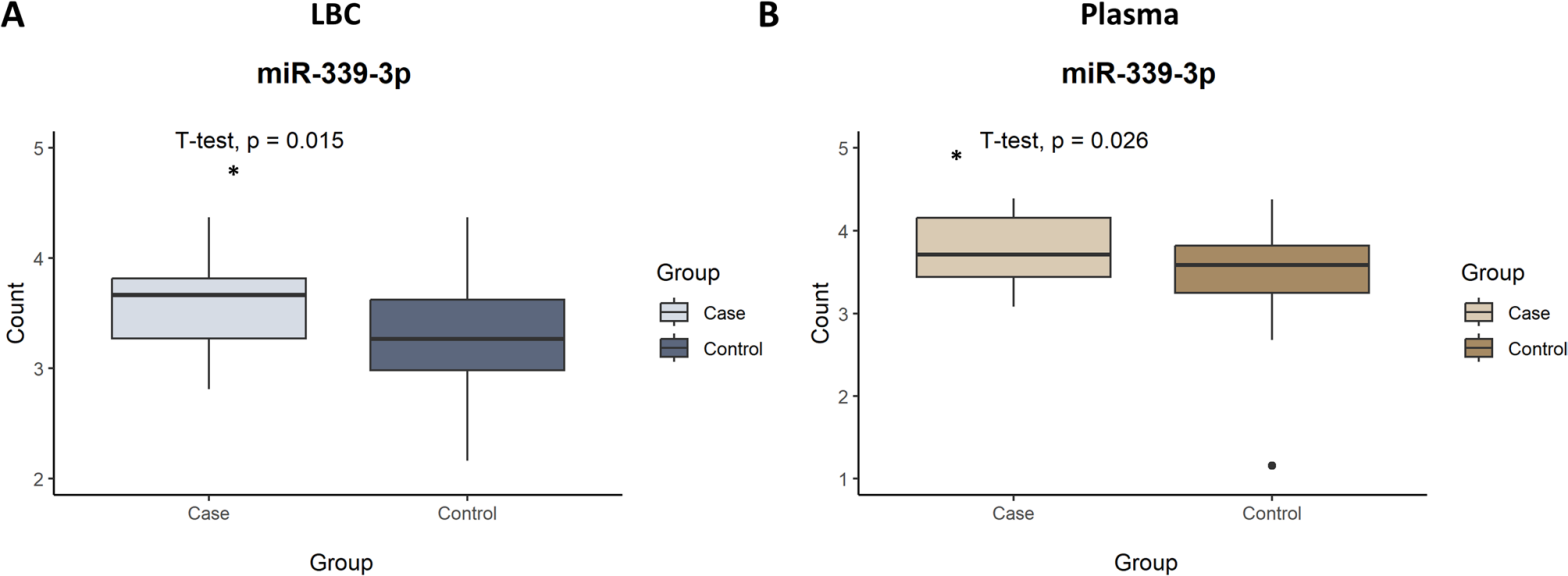
Box plot showing the differential expression of miR-339-3p between case and control groups in LBC (**A**) and plasma (**B**) samples. Student’s t-test, *p < 0.05.

Boxplots were generated to visualize the expression difference of miR-339-3p between case and control groups in both LBC (Fig. 3A) and plasma samples (Fig. 3B). Significant upregulation was observed in the case group for both sample types (*p* = 0.015 for LBC and *p* = 0.026 for plasma; Student’s t-test).

**Fig. 4:**
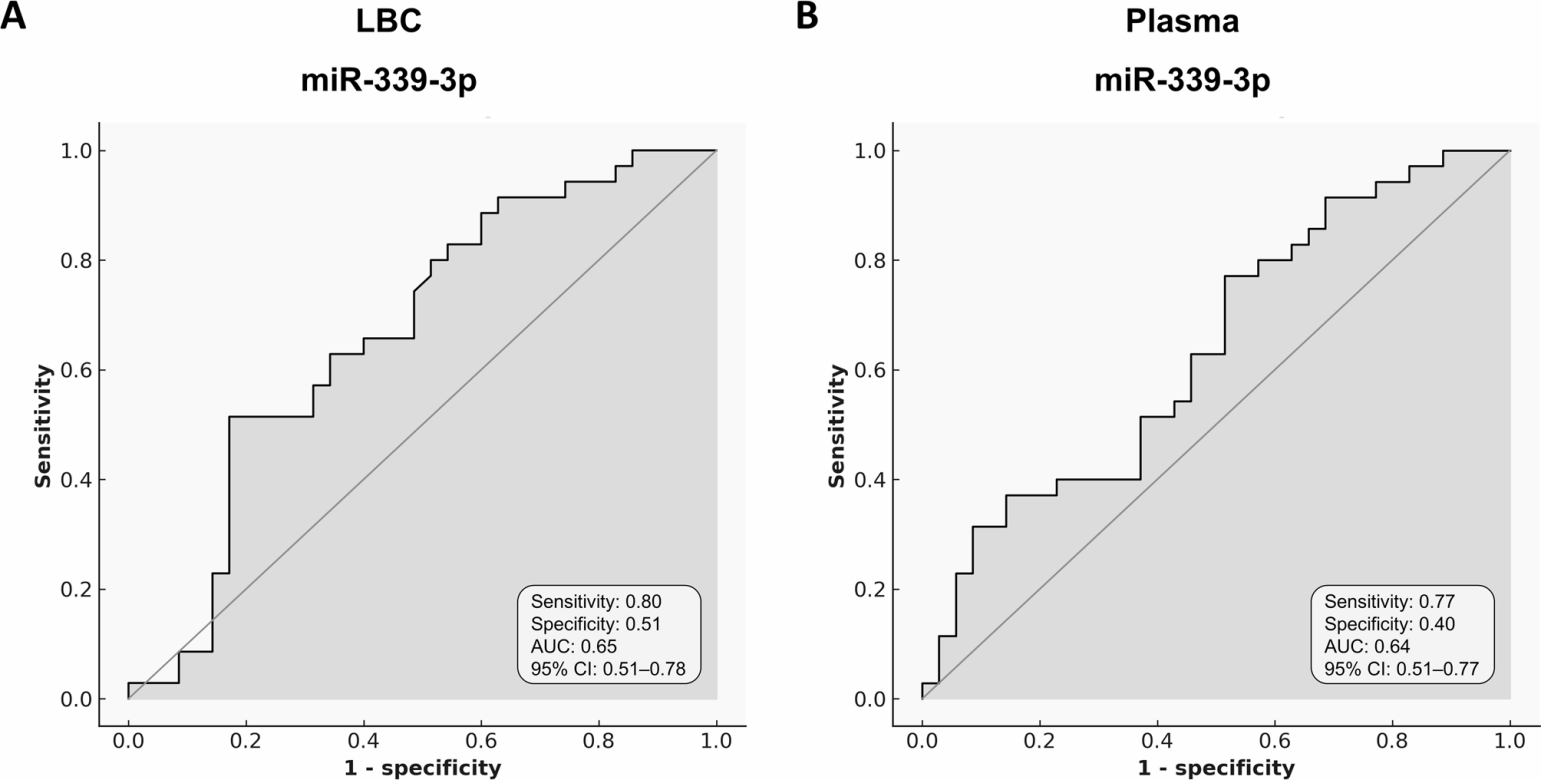
ROC curves for miR-339-3p in different biological matrices. Receiver operating characteristic (ROC) curves show the diagnostic performance of miR-339-3p in discriminating cases from controls in (**A**) liquid-based cytology (LBC) and (**B**) plasma samples.

To assess the predictive value of miR-339-3p in identifying high-grade cervical lesions, ROC curve analysis was conducted using both LBC and plasma samples. A minimum sensitivity threshold of 0.75 was predefined and fixed for this analysis. In LBC, miR-339-3p showed an area under the curve (AUC) of 0.65 (95% CI 0.51–0.78) (Fig. 4A), with sensitivity and specificity values of 0.80 and 0.51, respectively. In plasma, the AUC was 0.64 (95% CI 0.51–0.77) (Fig. 4B), with a sensitivity of 0.77 and specificity of 0.40. We also evaluated the correlation of miR-339-3p expression between LBC and plasma. Spearman’s rank analysis showed a very weak and non-significant correlation (ρ = 0.13, *p* = 0.25) (Supplementary Figure S1).

### Target prediction and pathway enrichment – miR-339-3p

**Fig. 5:**
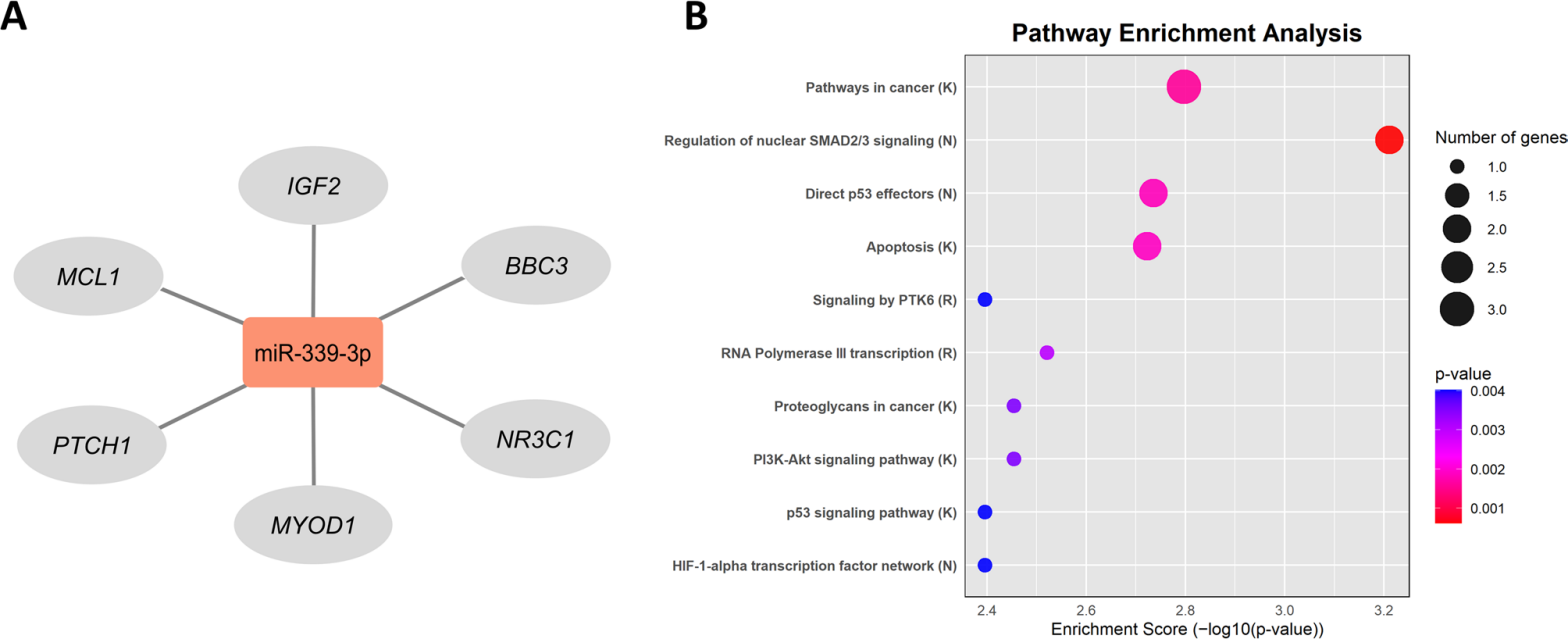
Target gene interaction and functional enrichment analyses for miR-339-3p. (**A**) Schematic representation of the interaction between miR-339-3p (red, overexpressed) and its predicted target genes (grey). (**B**) Top 10 significantly enriched pathways based on the target genes of miR-339-3p. Databases used include: Kyoto Encyclopedia of Genes and Genomes (K), National Cancer Institute (N), and Reactome (R). X-axis values represent statistical significance (− log₁₀(p-value)). *p* < 0.05.

In the target prediction analysis of miR-339-3p, 193 target genes were identified, of which six are directly associated with the disease studied (cervical cancer). Figure 5A illustrates the interaction between miR-339-3p and its predicted target genes (*IGF2*,* MCL1*,* PTCH1*,* MYOD1*,* NR3C1*, and *BBC3*).

Functional enrichment analysis was performed to further investigate the biological relevance of these targets. Figure 5B presents a dot plot displaying the top 10 significantly enriched pathways, derived from the predicted targets of miR-339-3p. Dot size corresponds to the number of genes involved in each pathway, while color intensity reflects statistical significance (p-value). Notably enriched pathways include cancer-related signaling, the PI3K-Akt signaling pathway, the p53 signaling pathway, and the role of proteoglycans in cancer. The genes corresponding to each of these pathways are available in Supplementary Table S3. Gene ontology analyses were also performed, revealing the top ten enriched biological processes and molecular functions of the target genes (Supplementary Figure S2).

## Discussion

This exploratory case–control study investigated miRNA expression in both local (LBC) and systemic (plasma) samples to identify candidate biomarkers associated with high-grade cervical lesions. Our study explored whether circulating miRNAs could complement existing screening tools by providing additional biologically relevant information through minimally invasive approaches.

Our results highlight miR-339-3p as a potential biomarker for high-grade cervical intraepithelial lesion (CIN 2/3), as it was consistently detected in both LBC and plasma samples. In LBC, miR-339-3p remained significantly associated with the case group even after the multivariate model, suggesting independent diagnostic potential. In plasma, although also significantly upregulated, its discriminative ability appeared more modest and dependent on the combination with other miRNAs.

The consistent detection of miR-339-3p across both matrices highlights its biological relevance. LBC captures local alterations in cervical epithelial cells^19^, while plasma reflects systemic molecular signals^10^. Consistently, the very weak and non-significant correlation between LBC and plasma levels of miR-339-3p (ρ = 0.13, *p* = 0.25) reinforces that these matrices represent distinct biological compartments. Rather than redundancy, this finding highlights their complementary value in biomarker discovery and supports the rationale of our dual-matrix approach. Despite this dual presence, miR-339-3p exhibited only moderate discriminatory performance (AUC < 0.70), indicating that it is unlikely to serve as a stand-alone biomarker. Nevertheless, it is notable that miR-339-3p achieved a sensitivity greater than 0.75 in both matrices, a threshold we predefined to address the known limitations in Pap smear sensitivity^7^. To further evaluate the robustness of these classification models, we performed a 5-fold cross-validation, which yielded an overall accuracy of approximately 60%, indicating only moderate discriminative performance and likely reflecting the limited sample size and exploratory nature of this study (Supplementary Tables S4 and S5).This supports its potential as a complementary marker to enhance diagnostic accuracy in cervical cancer screening.

An additional finding of interest was the observed association between current use of hormonal contraceptives and case status in both matrices. Although this variable was not the primary focus of our study, it is well established that hormonal regulation can modulate miRNA expression^20^. This association warrants further investigation to clarify potential interactions between hormonal factors and miRNA-based molecular markers in cervical carcinogenesis.

Members of the miR-339 family (miR-339-3p and miR-339-5p) have been implicated in hormone-related gynecological cancers, particularly breast and ovarian tumors. In breast cancer, miR-339-5p has been described as a tumor suppressor. Wu et al. reported that its downregulation promotes proliferation and invasion via BCL6 targeting^21^. Additionally, Feng et al. demonstrated that miR-339-5p is integrated into estrogen-regulated pathways through the MAFG-AS1/miR-339-5p/CDK2 axis, contributing to tumor progression and endocrine resistance in estrogen receptor–positive breast cancer^22^. In ovarian cancer, Shan et al. showed that miR-339-5p inhibits migration and invasion through targeting NACC1 and BCL6^23^, while Zhou et al. reported differential expression of miR-339-5p in exosomal miRNA profiles from ovarian cancer patients^24^. Collectively, these findings suggest that miR-339 expression is modulated by hormonal pathways in multiple gynecological malignancies. This may provide a biological context to further explore the observed association between miR-339-3p levels and current hormonal contraceptive use in cervical precursor lesions. In this regard, hormonal status could represent an important modifier of miRNA-based biomarker performance, and its integration into future screening models may help refine risk stratification and improve the accuracy of molecular triage strategies. While most studies in gynecological cancers have focused on miR-339-5p, miR-339-3p has been independently described as a tumor suppressor in other malignancies, including in melanoma^17^, colorectal cancer^18^, nasopharyngeal carcinoma^25^, lung^16^, and prostate cancer^26^, further highlighting its broad biological relevance. The biological behavior of the miR-339 family appears to be highly context-dependent.

In our study, we observed upregulation of miR-339-3p in both LBC and plasma samples from women with high-grade cervical lesions. This finding contrasts with the tumor-suppressive profile previously reported for miR-339-3p in other malignancies, and also diverges from the predominantly suppressive role described for miR-339-5p in hormone-driven gynecological cancers. These differences may reflect the distinct molecular environment of HPV-associated cervical cancer. High-risk HPV oncoproteins such as E6 and E7 are known to profoundly alter host miRNA expression^27^, potentially contributing to the observed modulation of miR-339-3p in this context. Supporting its biological relevance, functional enrichment analysis of predicted targets of miR-339-3p revealed significant involvement in cancer-related pathways, particularly the p53 signaling pathway, which is critically disrupted in HPV-associated lesions^28^. The p53 pathway plays an essential role in cell cycle control and apoptosis, processes frequently affected by high-risk HPV types through the E6 oncoprotein, which promotes p53 degradation^29^.

Among the predicted targets of miR-339-3p identified in our analysis, MCL1 and BBC3 (PUMA) are directly related to the p53 pathway. MCL1 is an antiapoptotic protein frequently overexpressed in tumors^30^, while BBC3 is a key proapoptotic factor activated by p53^31^. Notably, MCL1 has also been experimentally validated as a direct target of miR-339-3p in melanoma^17^, supporting its relevance as a potential regulatory node. Given the upregulation of miR-339-3p in both LBC and plasma samples in our study population, the regulation of these targets in the specific context of HPV-induced cervical cancer remains to be experimentally validated, as it may differ from mechanisms described in other tumor types. As this is an exploratory study based on in silico predictions, further functional analyses will be necessary to confirm these findings.

Beyond its biological relevance, the potential clinical utility of miR-339-3p warrants further investigation, particularly in the evolving landscape of cervical cancer screening. Its consistent detection in both LBC and plasma suggests that it could complement existing approaches, either as part of broader molecular investigations or as an adjunct to HPV testing^11,15,32^. As molecular HPV testing becomes the preferred method for primary screening in many countries, there is growing interest in complementary biomarkers to refine the triage of hr-HPV-positive women^33^. Around 15–20% of screened women test positive for hr-HPV, but most do not have clinically significant lesions, often leading to unnecessary colposcopies and follow-up procedures^34^. In this context, miRNAs such as miR-339-3p could serve as additional molecular tools to help identify women with underlying CIN 2/3. Notably, miRNA analysis could be performed using the same LBC sample already collected for HPV testing^35^, supporting the rationale for integrating miRNA assays into cytology-based workflows^19^. In parallel, plasma may offer additional non-invasive options, particularly for populations with limited access to cervical sampling or for longitudinal monitoring of lesion progression and treatment response^11^. Specifically, miR-339-3p could add value at three clinical decision points: (I) triage of hr-HPV–positive women to reduce unnecessary colposcopies; (II) reflex testing in equivocal cytology (ASC-US/LSIL) to refine risk assessment; and (III) longitudinal surveillance after treatment of CIN2/3 to monitor persistence or recurrence. These findings support further research into miR-339-3p as part of more personalized and dynamic cervical cancer screening strategies.

Beyond cervical disease, circulating miRNAs have been increasingly studied as minimally invasive biomarkers in several malignancies^13^. Recent reviews have highlighted their role in bladder, kidney, and prostate cancers, reinforcing the broad potential of miRNAs in liquid biopsy for early detection and diagnosis^36^. In parallel, multi-fluid strategies have demonstrated clinical utility in other contexts, such as prostate cancer, where blood- and urine-based assays have improved early detection and risk stratification^37^. Our findings contribute to this broader approach by demonstrating that the combined analysis of LBC and plasma offers complementary information and exemplifies how integrative, multi-fluid strategies can advance minimally invasive biomarker development in cervical disease.

To our knowledge, this is the first study to investigate and directly compare miRNA expression in both liquid-based cytology (LBC) and plasma samples from women with high-grade cervical intraepithelial lesions. This dual-matrix approach allowed the identification of miR-339-3p as a shared and consistently upregulated biomarker, reinforcing its biological relevance and potential for clinical application in minimally invasive screening strategies. The use of the NanoString nCounter platform ensured robust and reproducible quantification of a broad panel of miRNAs, and the integration of statistical and bioinformatics analyses added depth to the interpretation of the data. Furthermore, the association of miR-339-3p with cancer-related pathways such as PI3K-Akt and p53 signaling strengthens the mechanistic plausibility of its involvement in cervical carcinogenesis. Collectively, these features position miR-339-3p as a promising candidate biomarker that warrants further investigation and validation in larger, independent cohorts.

Despite our findings, our study has some limitations, including a relatively small sample size, which may have limited statistical power and contributed to wide confidence intervals in some regression models. Furthermore, our results should be considered exploratory and hypothesis-generating rather than definitive. External validation in larger, independent cohorts will be essential before any clinical translation of these findings can be considered. Also, due to the cross-sectional design, causal relationships between miRNA expression and lesion progression could not be established. Additionally, further experimental assays of miR-339-3p molecular targets and pathways are needed to validate our bioinformatic analyses. Finally, although we observed a statistically significant association between hormonal contraceptive use and miRNA expression, this was not a primary outcome and should be explored in future studies.

**Fig. 6:**
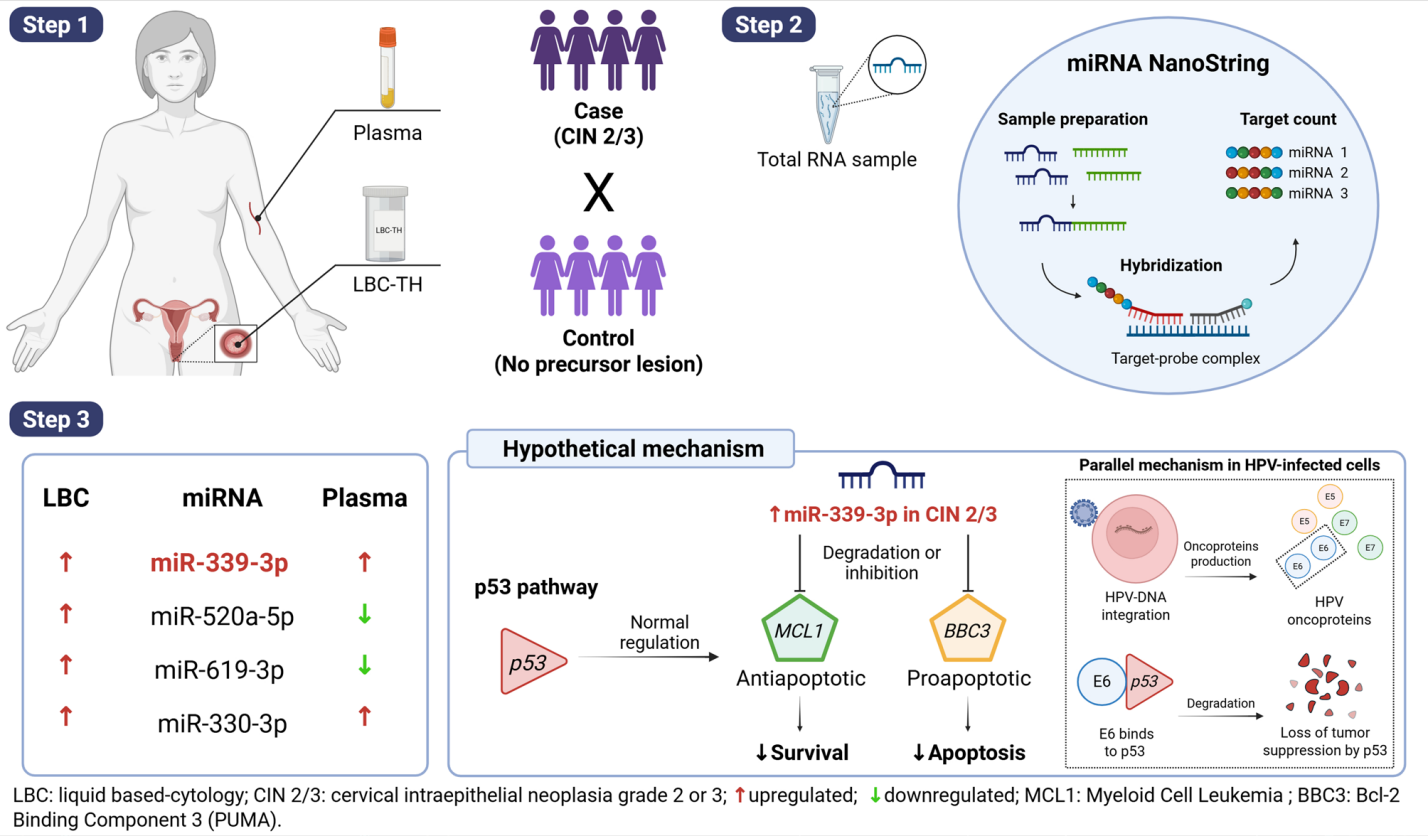
Graphical summary of miR-339-3p as a shared biomarker in LBC and plasma in CIN 2/3. Step 1: LBC and plasma samples were collected from women with CIN 2/3 (cases) and women with normal cytology (controls). Step 2: Total RNA was extracted, and miRNA expression was analyzed using the NanoString platform. Step 3: Four differentially expressed miRNAs were identified in LBC, with miR-339-3p being the only one consistently upregulated in both LBC and plasma. Functional analysis suggests that miR-339-3p may modulate the p53 pathway by targeting MCL1 and BBC3, contributing to apoptotic dysregulation in HPV-infected cells. Created with BioRender by the author.

In summary, this study suggests that miR-339-3p may be a relevant biomarker for CIN 2/3, as it was the only miRNA consistently upregulated in both LBC and plasma samples. Functional analysis indicated its potential involvement in the p53 signaling pathway, particularly through predicted regulation of key apoptosis-related genes such as MCL1 and BBC3, suggesting a role in modulating apoptosis in HPV-related cervical lesions (Fig. 6**)**.

In conclusion, our findings provide the first evidence of miR-339-3p upregulation in both LBC and plasma samples from women with high-grade cervical lesions, suggesting its potential relevance in the context of HPV-associated cervical cancer. While its performance alone was limited for diagnostic purposes, its consistent detection across two distinct biological matrices and predicted association with key regulatory pathways support further investigation. As this is an exploratory study, validation in larger cohorts and functional analyses will be essential to better define the role of miR-339-3p and assess its potential clinical utility in cervical cancer screening strategies.

## Methods

### Study population and sample collection

The present cross-sectional study included 70 women, with paired liquid-based cytology (LBC) and plasma samples collected from each participant at the Prevention Department of Barretos Cancer Hospital from November 2021 to August 2023. The study was approved by the Research Ethics Committee of the Barretos Cancer Hospital, Brazil (approval no. 3.926.525). All procedures were conducted in accordance with the Declaration of Helsinki and in compliance with all relevant institutional, national, and international regulations. Written informed consent was obtained from every participant (or their legal guardian, where applicable), including explicit permission for the publication of anonymised data. No identifying images or other personal information are presented in this article; all clinical and laboratory data were stored in an encrypted, password-protected database accessible only to the study team.

Eligible participants were between 25 and 64 years old, with no history of cervical precursor lesions, no previous cervical procedures, or prior cancer diagnoses. The case group included 35 women with histologically confirmed high-grade cervical intraepithelial lesion (CIN 2/3), confirmed by histopathological examination of the excised tissue obtained through the Loop Electrosurgical Excision Procedure (LEEP). All patients in the case group were tested for hr-HPV. The control group also comprised 35 women, selected based on negative results for both Pap smear cytology and high-risk HPV testing, performed using the Cobas ×480 system (Roche Molecular Systems, Pleasanton, CA, USA)^38^. Patients were matched by age (± 2 years).

LBC cervical samples were obtained from each participant immediately before LEEP (case group) and Pap smear (control group). Cervical samples were obtained using a Cervex-Brush (Rovers Medical Devices, North Brabant, Netherlands – cat. no. 36825G). Two smears were taken from each participant: one preserved in SurePath Preservative Fluid (Becton-Dickinson, Franklin Lakes, NJ, USA – cat. no. 491337) for routine cytological and HPV testing, and another preserved in ThinPrep Pap Test medium (Hologic, Bedford, MA, USA – cat. no. 31845) for subsequent miRNA expression analysis. Peripheral blood was collected into EDTA-containing tubes for plasma separation. All samples were processed and stored at the Barretos Cancer Hospital Biobank as described^39^.

### RNA isolation from biological matrices

Cervical LBC and plasma samples were initially processed and stored at − 80 °C within 4 h (LBC) and 2 h (plasma) after collection. Total RNA was subsequently extracted using the miRNeasy Mini Kit (Qiagen, Hilden, GER; cat. nº 217004) for LBC and the miRNeasy Serum/Plasma Kit (Qiagen, Hilden, GER; cat. nº 217184) for plasma, following the manufacturer’s protocols. The extracted RNA was stored at − 80 °C until further use. RNA concentration and purity were assessed using the NanoDrop 2000/2000c Spectrophotometer (Thermo Fisher Scientific, Waltham, MA, USA).

### NanoString nCounter system assays

miRNA expression profiling was conducted using the nCounter Human v3A miRNA Expression Assay Panel (NanoString Technologies, Seattle, WA, USA – cat. no. CSO-MIR3-12), following the manufacturer’s instructions. This platform enables the quantification of 798 human miRNAs. Briefly, 100 ng of total RNA was hybridized with Reporter CodeSet and Capture ProbeSet for 24 h at 65 °C, allowing miRNA-specific tag binding. Hybridized samples were then processed using the NanoString PrepStation and transferred into a nCounter cartridge. Subsequently, data were acquired using the nCounter^®^ Digital Analyzer, with image collection performed across 555 fields of view (FOVs).

### Statistical analysis

Analyses were conducted using R version 4.4 in RStudio 2024^40^. Differential expression analysis of miRNAs was performed using the limma package from Bioconductor. A p-value threshold of < 0.05 was adopted to determine statistical significance between groups. Raw count data from the NanoString platform were normalized using the NanoStringNorm package based on the lowest coefficient of variation and fold change estimation. To assess the diagnostic performance of differentially expressed miRNAs, receiver operating characteristic (ROC) curves were generated, and the area under the curve (AUC) was calculated using the pROC and ROCR packages. Sensitivity and specificity analyses were also carried out to evaluate the discriminatory ability of selected miRNAs, and all analyses were performed using the entire dataset. The correlation between miR-339-3p expression levels in LBC and plasma was assessed using Spearman’s rank correlation coefficient. For group prediction (case/control), a multivariate logistic regression model was used, applying the backward selection criterion to identify significant variables. Model performance was evaluated using 5-fold cross-validation, and the overall agreement between predicted and observed classifications was calculated. Statistical significance was set at *p* < 0.05.

Table S6 (Supplementary Information) describes the reference (housekeeping) miRNAs used for normalization in different matrices.

### MicroRNA target prediction

To evaluate the biological role of miRNAs identified as potential biomarkers, target prediction analysis was performed using the online platform miRDIP (http://ophid.utoronto.ca/mirDIP/), considering only the top 1% of predicted interactions (score class: very high), based on integrated evidence from multiple databases. Predicted targets were filtered to include those supported by at least three of the following sources: DIANA, microRNA.org, RNA22, RNAHybrid, and TargetScan. The resulting gene list was then imported into Cytoscape^41^ for integration, visualization, and analysis of regulatory networks.

### Biological pathway enrichment

Pathway enrichment analysis was conducted using the ReactomeFI plugin within Cytoscape, incorporating selected biological pathways and interaction networks from Reactome and additional data sources. For this analysis, only genes associated with cervical cancer, as annotated in the Cancer Gene Index by the National Cancer Institute, were selected via the Load Cancer Index feature of ReactomeFIViz. In addition to pathway analysis, Gene Ontology (GO) enrichment was also performed using the same plugin, covering the categories of Biological Process, Molecular Function, and Cellular Component. Enriched pathways and GO terms were considered significant if they had a false discovery rate (FDR) < 0.05.

## Supplementary Information

Below is the link to the electronic supplementary material.

Supplementary Material 1

## Data Availability

The data that support the findings of this study are not openly available due to reasons of sensitivity and are available from the corresponding author upon reasonable request. Data are located in controlled access data storage at Teaching and Research Institute (Barretos Cancer Hospital).
