# Supplementary material for: Exploring shared microRNA profiles in liquid-based cytology and plasma as biomarkers of high-grade intraepithelial lesions
Source: Sci Rep. 2025 Dec 2;16:828. doi: 10.1038/s41598-025-30514-3 (PMC12780091; doi:10.1038/s41598-025-30514-3)
Supplement: Supplementary file 1 — Supplementary Material 1 [file 41598_2025_30514_MOESM1_ESM.docx]

**Shared microRNA profiles in liquid-based cytology (LBC) and plasma samples: exploring biomarkers for high-grade intraepithelial lesion**

Stéphanie Calfa^1^*, Ana Julia Aguiar de Freitas^1^, Rhafaela Lima Causin^1^, Welinton Hirai^1^, Júlio César Possati-Resende^2^, Ricardo dos Reis^3^, Rui Manuel Reis^1,4^, and Márcia Maria Chiquitelli Marques^1^*.

| Table S1. Differentially expressed miRNAs were identified between the control and case groups in plasma samples. | | | |
| --- | --- | --- | --- |
| miRNA | logFC | Expression | p-value |
| hsa-miR-144-3p | -2.82 | downregulated | <0.001 |
| hsa-miR-1268a | 0.48 | upregulated | <0.001 |
| hsa-miR-451a | -2.64 | downregulated | <0.001 |
| hsa-miR-1269a | 0.27 | upregulated | <0.001 |
| hsa-miR-223-3p | 1.96 | upregulated | <0.001 |
| hsa-miR-1255b-5p | 0.29 | upregulated | <0.01 |
| hsa-miR-582-5p | 0.35 | upregulated | <0.01 |
| ­hsa-miR-320e | 0.84 | upregulated | <0.01 |
| hsa-miR-579-5p | 0.26 | upregulated | <0.01 |
| hsa-miR-1302 | -0.22 | downregulated | <0.01 |
| hsa-miR-1293 | 0.27 | upregulated | <0.01 |
| hsa-miR-4425 | 0.24 | upregulated | <0.01 |
| hsa-miR-1910-5p | -0.46 | downregulated | <0.01 |
| hsa-miR-4516 | 0.59 | upregulated | <0.01 |
| hsa-miR-1915-3p | 0.47 | upregulated | <0.01 |
| hsa-miR-346 | 0.21 | upregulated | <0.01 |
| hsa-miR-574-5p | 1.16 | upregulated | <0.01 |
| hsa-miR-1297 | 0.21 | upregulated | <0.01 |
| hsa-miR-320a | 0.31 | upregulated | <0.01 |
| hsa-miR-601 | 0.47 | upregulated | <0.05 |
| hsa-miR-330-3p | 0.24 | upregulated | <0.05 |
| hsa-miR-2116-5p | 0.28 | upregulated | <0.05 |
| hsa-miR-596 | 0.26 | upregulated | <0.05 |
| hsa-miR-612 | 0.26 | upregulated | <0.05 |
| hsa-miR-640 | 0.23 | upregulated | <0.05 |
| hsa-miR-126-3p | -0.74 | downregulated | <0.05 |
| hsa-miR-548e-3p | -0.18 | downregulated | <0.05 |
| hsa-miR-517c-3p+hsa-miR-519a-3p | -0.15 | downregulated | <0.05 |
| hsa-miR-497-5p | -0.28 | downregulated | <0.05 |
| hsa-miR-516a-5p | 0.23 | upregulated | <0.05 |
| hsa-miR-510-5p | 0.24 | upregulated | <0.05 |
| hsa-miR-1281 | -0.20 | downregulated | <0.05 |
| hsa-miR-379-5p | 0.26 | upregulated | <0.05 |
| hsa-miR-487a-3p | -0.15 | downregulated | <0.05 |
| hsa-miR-1245b-5p | 0.19 | upregulated | <0.05 |
| hsa-miR-325 | 0.19 | upregulated | <0.05 |
| hsa-miR-135a-5p | -0.58 | downregulated | <0.05 |
| hsa-miR-483-5p | 0.27 | upregulated | <0.05 |
| hsa-miR-204-5p | 0.36 | upregulated | <0.05 |
| hsa-miR-1296-5p | -0.22 | downregulated | <0.05 |
| hsa-miR-339-3p | 0.23 | upregulated | <0.05 |
| hsa-miR-642a-3p | 0.65 | upregulated | <0.05 |
| hsa-miR-1305 | 0.20 | upregulated | <0.05 |
| hsa-miR-340-5p | 0.42 | upregulated | <0.05 |
| hsa-miR-1185-2-3p | -0.19 | downregulated | <0.05 |
| hsa-miR-520a-5p | 0.22 | upregulated | <0.05 |
| hsa-miR-9-5p | 0.35 | upregulated | <0.05 |
| hsa-miR-514a-5p | -0.27 | downregulated | <0.05 |
| hsa-miR-150-5p | -0.87 | downregulated | <0.05 |
| hsa-miR-1322 | 0.17 | upregulated | <0.05 |
| hsa-miR-6511a-3p | 0.25 | upregulated | <0.05 |
| hsa-miR-619-3p | 0.28 | upregulated | <0.05 |
| hsa-miR-595 | 0.26 | upregulated | <0.05 |
| hsa-miR-877-5p | 0.21 | upregulated | <0.05 |
| hsa-miR-1269b | 0.23 | upregulated | <0.05 |
| hsa-miR-190b | 0.26 | upregulated | <0.05 |
| hsa-miR-548c-5p+hsa-miR-548o-5p+hsa-miR-548am-5p | 0.25 | upregulated | <0.05 |
| FC: fold-change; hsa: homo sapiens. | | | |

| Table S2. Differentially expressed miRNAs were identified between the control and case groups in plasma samples. | | | |
| --- | --- | --- | --- |
| miRNA | **FC** | **Expression** | **p-value** |
| hsa-miR-6724-5p | 1.29 | upregulated | <0.001 |
| hsa-miR-556-3p | 1.36 | upregulated | <0.01 |
| hsa-miR-520d-5p+hsa-miR-527+hsa-miR-518a-5p | 1.30 | upregulated | <0.01 |
| hsa-miR-553 | -1.27 | downregulated | <0.01 |
| hsa-miR-548h-5p | 1.32 | upregulated | <0.01 |
| hsa-miR-619-3p | -1.32 | downregulated | <0.01 |
| hsa-miR-5010-3p | -1.22 | downregulated | <0.05 |
| hsa-miR-532-5p | -1.29 | downregulated | <0.05 |
| hsa-miR-4787-5p | -1.24 | downregulated | <0.05 |
| hsa-miR-630 | -1.28 | downregulated | <0.05 |
| hsa-miR-652-5p | -1.28 | downregulated | <0.05 |
| hsa-miR-27a-3p | -1.21 | downregulated | <0.05 |
| hsa-miR-339-3p | 1.30 | upregulated | <0.05 |
| hsa-miR-1323 | -1.20 | downregulated | <0.05 |
| hsa-miR-518d-3p | -1.37 | downregulated | <0.05 |
| hsa-miR-1226-3p | -1.23 | downregulated | <0.05 |
| hsa-miR-4531 | -1.23 | downregulated | <0.05 |
| hsa-miR-4461 | -1.16 | downregulated | <0.05 |
| hsa-miR-873-5p | -1.33 | downregulated | <0.05 |
| hsa-miR-330-5p | -1.28 | downregulated | <0.05 |
| hsa-miR-138-5p | -1.22 | downregulated | <0.05 |
| hsa-miR-520a-5p | -1.18 | downregulated | <0.05 |
| hsa-miR-4524a-5p | 1.30 | upregulated | <0.05 |
| hsa-miR-935 | 1.25 | upregulated | <0.05 |
| hsa-miR-515-3p | 1.20 | upregulated | <0.05 |
| hsa-miR-324-5p | -1.32 | downregulated | <0.05 |
| hsa-miR-3918 | -1.29 | downregulated | <0.05 |
| hsa-miR-141-3p | -1.23 | downregulated | <0.05 |
| hsa-miR-330-3p | 1.27 | upregulated | <0.05 |
| hsa-miR-499a-5p | -1.23 | downregulated | <0.05 |
| hsa-miR-769-5p | -1.18 | downregulated | <0.05 |
| hsa-miR-532-3p | 1.16 | upregulated | <0.05 |
| hsa-miR-6721-5p | -1.17 | downregulated | <0.05 |
| FC: fold-change; hsa: homo sapiens. | | | |

| **Table S3.** Enrichment analysis of pathways with their respective genes involved in miR-339-3p. | | | |
| --- | --- | --- | --- |
| **Pathways** | **FDR** | **n** | **Gene** |
| Regulation of nuclear SMAD2/3 signaling(N) | 0,024 | 2 | *MYOD1, NR3C1* |
| Pathways in cancer(K) | 0.024 | 3 | *PTCH1, IGF2, BBC3* |
| Direct p53 effectors(N) | 0.024 | 2 | *BBC3, MCL1* |
| Apoptosis(K) | 0.024 | 2 | *BBC3, MCL1* |
| RNA Polymerase III transcription(R) | 0.024 | 1 | *NR3C1* |
| Proteoglycans in cancer(K) | 0.025 | 2 | *PTCH1, IGF2* |
| PI3K-Akt signaling pathway(K) | 0.025 | 2 | *IGF2, MCL1* |
| Signaling by PTK6(R) | 0.025 | 1 | *NR3C1* |
| HIF-1-alpha transcription factor network(N) | 0.032 | 1 | *MCL1* |
| p53 signaling pathway(K) | 0.036 | 1 | *BBC3* |
| K: Kyoto Encyclopedia of Genes and Genomes (KEGG); R: Reactome; N: National Cancer Institute (NCI); N: number of genes involved. | | | |


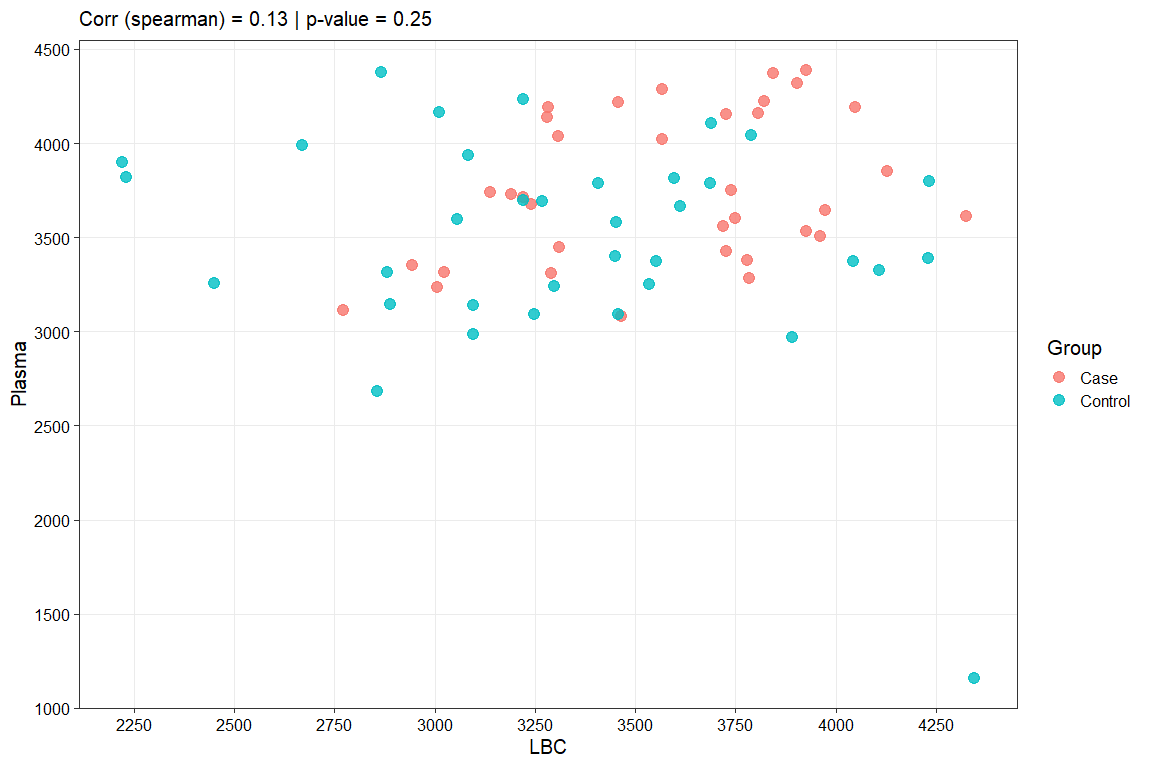


**Figure S1.** Scatterplot of miR-339-3p expression in paired LBC and plasma samples. Scatterplot showing the relationship between miR-339-3p expression levels measured in liquid-based cytology (LBC) and plasma. Each dot represents one participant (red = CIN2/3 cases; blue = controls). Spearman’s rank correlation analysis indicated a very weak and non-significant association between LBC and plasma levels (ρ = 0.13, p = 0.25).

**
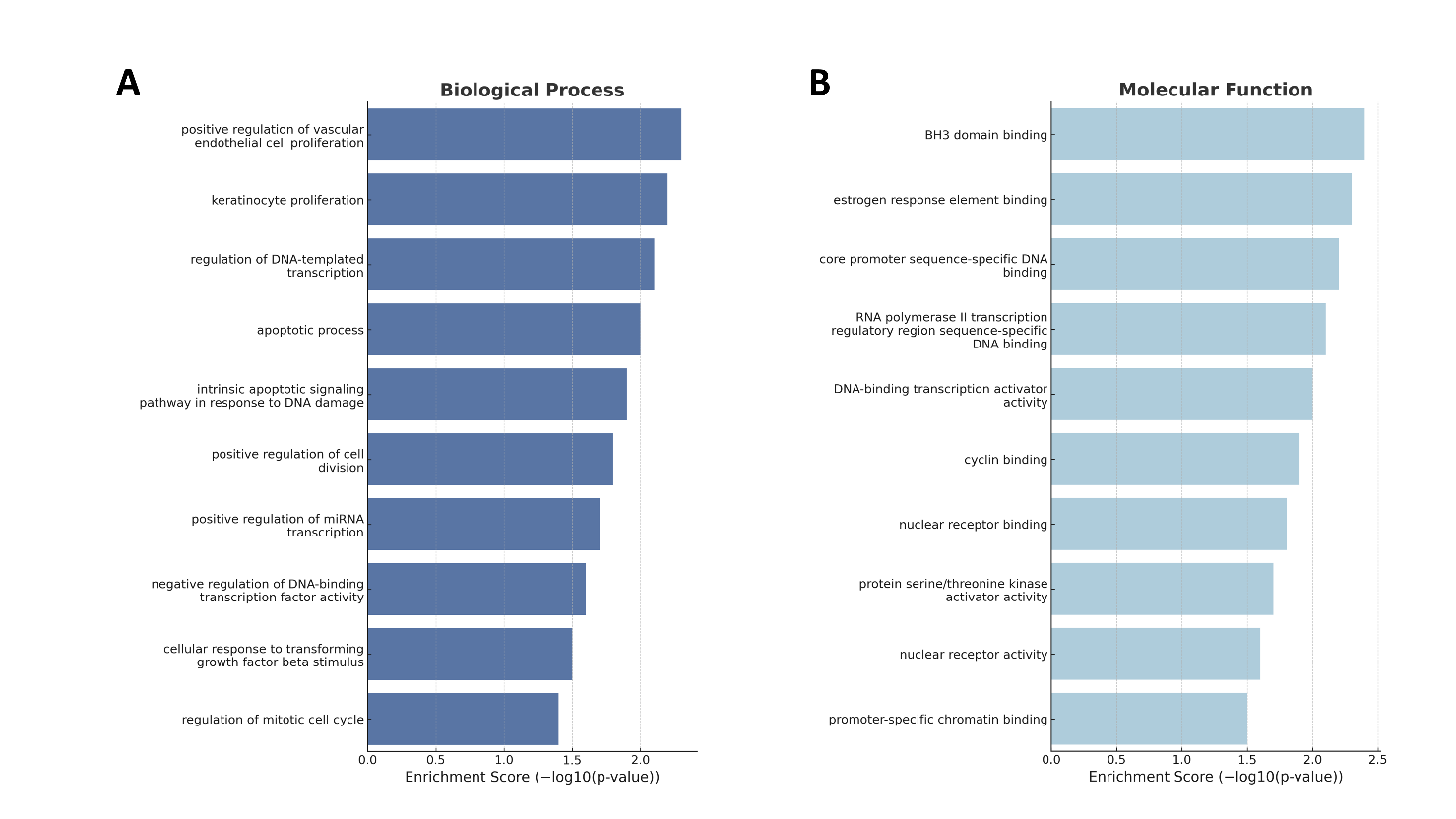
**

**Figure S2.** Functional enrichment analysis of predicted miR-339-3p target genes.
(**A**) Enriched biological processes and (**B**) molecular functions identified through gene ontology (GO) analysis of the predicted miR-339-3p target genes. The x-axis represents the enrichment score (−log₁₀(p-value)). Only GO terms with statistical significance (p < 0.05) are displayed. Among the top enriched biological processes are positive regulation of vascular endothelial cell proliferation, keratinocyte proliferation, regulation of transcription, apoptotic processes, and DNA damage response. Enriched molecular functions include BH3 domain binding, estrogen response element binding, sequence-specific DNA binding, transcriptional regulation, and nuclear receptor activity.

| **Table S4.** Multivariate Logistic Regression Model (Outcome: group \| Predictors: miR-339, Anti) with 5-Fold Cross-Validation using the LBC dataset. | | | | |
| --- | --- | --- | --- | --- |
| **Model** | | **Data** | | |
|  |  | **Case** | **Control** | **Total** |
| Case | Count | 20 | 12 | 32 |
|  | Column % | 57.1% | 34.3% |  |
| Control | Count | 15 | 23 | 38 |
|  | Column % | 42.9% | 65.7% |  |
| Total | Count | 35 | 35 | 70 |
| The table summarizes the predicted versus observed classifications obtained after cross-validation. “Count” indicates the number of samples correctly or incorrectly classified in each category, and “Column %” represents the proportion within each observed group. The overall agreement (accuracy) between predicted and actual classes was 61.43%. | | | | |

| **Table S5.** Multivariate Logistic Regression Model (Outcome: group \| Predictors: miR-339, Anti) with 5-Fold Cross-Validation using the Plasma dataset. | | | | |
| --- | --- | --- | --- | --- |
| **Model** | | **Data** | | |
|  |  | **Case** | **Control** | **Total** |
| Case | Count | 18 | 10 | 28 |
|  | Column % | 51.4% | 28.6% |  |
| Control | Count | 17 | 25 | 42 |
|  | Column % | 48.6% | 71.4% |  |
| Total | Count | 35 | 35 | 70 |
| The table summarizes the predicted versus observed classifications obtained after cross-validation. “Count” indicates the number of samples correctly or incorrectly classified in each category, and “Column %” represents the proportion within each observed group. The overall agreement (accuracy) between predicted and actual classes was 61.43%. | | | | |

| **Table S6.** Reference microRNAs for LBC and plasma samples analysis. | |
| --- | --- |
| **Specimen** | **Housekeeping** |
| **LBC** | hsa-miR-1257 |
|  | hsa-miR-188-5p |
|  | hsa-miR-665 |
|  | hsa-miR-3690 |
|  | hsa-miR-585-3p |
|  | hsa-miR-3147 |
|  | hsa-miR-607 |
|  | hsa-miR-183-5p |
|  | hsa-miR-296-3p |
|  | hsa-miR-765 |
| **Plasma** | hsa-miR-584-5p |
|  | hsa-miR-3180 |
|  | hsa-miR-378i |
|  | hsa-miR-525-5p |
|  | hsa-miR-183-5p |
|  | hsa-miR-34a-5p |
|  | hsa-miR-25-5p |
|  | hsa-miR-887-5p |
|  | hsa-miR-3614-5p |
|  | hsa-miR-767-5p |
| This table presents the reference (housekeeping) miRNAs used for normalization in different types of matrices, including liquid-based cytology (LBC) and plasma. The normalization method used was Low Coefficient of Variation (Low CV). | |
